# Primary Erythrocytosis (Polycythaemia) in a Neutered Male Dog Presenting With Right Hindlimb Lameness

**DOI:** 10.1002/vms3.71320

**Published:** 2026-11-03

**Authors:** Robyn Belch, Perrine Henry, Marisa Ferreira, Albert Aguilera Padros, Maria Lazaro Munoz, Ben Lloyd‐Bradley, Ingrid Isaac, Efa Llewellyn, Aran Nagendran

**Affiliations:** ^1^ Veterinary Internal Medicine Service Hospital for Small Animals Royal (Dick) School of Veterinary Studies Easter Bush Campus, University of Edinburgh Edinburgh UK; ^2^ Veterinary Neurology/Neurosurgery Department Hospital for Small Animals Royal (DIck) School of Veterinary Studies, University of Edinburgh Easter Bush Campus, University of Edinburgh Edinburgh UK; ^3^ Veterinary Emergency and Critical Care Department Hospital for Small Animals Royal (Dick) School of Veterinary Studies Easter Bush Campus, University of Edinburgh Edinburgh UK; ^4^ Veterinary Diagnostic Imaging Department Hospital for Small Animals Royal (Dick) School of Veterinary Studies Easter Bush Campus, University of Edinburgh Edinburgh UK

**Keywords:** canine, erythrocytosis, hydroxyurea, hypercoagulable, polydipsia, thrombosis

## Abstract

A 2‐year‐old male entire Cocker Spaniel was referred for investigation of progressive right hindlimb lameness and polyuria and polydipsia. Investigations conducted by the primary care veterinary surgeon revealed erythrocytosis (haematocrit of 78%, reference interval: 37%–55%) and urine concentration consistent with polyuria and polydipsia (specific gravity 1.014). Upon referral, physical examination revealed a dark red oral mucosa and right hindlimb lameness, characterised by intermittent non‐weight bearing of the limb when standing. Neurological examination only revealed a mild short‐strided gait, and orthopaedic examination was unremarkable. Secondary causes of erythrocytosis were excluded by normal arterial blood gas, low erythropoietin levels and normal thoracic and abdominal imaging, resulting in a diagnosis of primary erythrocytosis (PE). Focal ultrasonography identified a right external iliac venous thrombus, suspected to be the cause of the right hindlimb lameness and presumed to be associated with the PE. Initial management involved repeated phlebotomies, followed by long‐term treatment with hydroxyurea (31 mg/kg twice weekly) and rivaroxaban (2 mg/kg once daily). At the 5‐month recheck, the dog was asymptomatic, with resolution of the iliac thrombus and a normal haematocrit. This case highlights an unusual presentation of PE in a dog, not previously described in the veterinary literature, and re‐examines the discussion on the optimal management of this rare condition, with a focus on the necessity of antithrombotic therapy.

## Graphical abstract

A young dog presented with progressive unilateral hindlimb lameness and marked erythrocytosis. Diagnostic investigations excluded secondary causes and confirmed primary erythrocytosis. Targeted vascular ultrasonography identified a right external iliac venous thrombus as the suspected cause of lameness. Management with phlebotomy, hydroxyurea and rivaroxaban resulted in resolution of the thrombus, normalisation of haematocrit and complete clinical recovery. This case highlights venous thrombosis as an unusual but important cause of lameness in dogs with erythrocytosis.

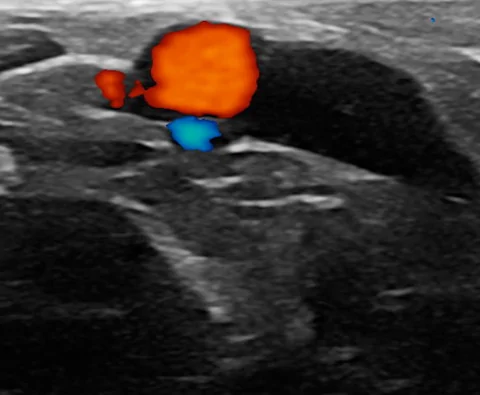

## 1 Introduction

Erythrocytosis refers to an increased haematocrit, which may result from either an increase in red blood cells or a decrease in plasma volume (Ettinger and Feldman 2016). Erythrocytosis is classified into three categories: ‘relative’ erythrocytosis (e.g., dehydration and splenic contraction); ‘secondary’ erythrocytosis, which is seen in disorders resulting in an exaggerated release of erythropoietin, occurring either appropriately in response to tissue hypoxia (e.g., cardiac and respiratory diseases) or inappropriately (e.g., secondary to renal hypoxia in some specific renal conditions or to neoplasia); and finally, ‘primary’ erythrocytosis, which refers to excessive red blood cell production that is independent of erythropoietin and instead results from an intrinsic bone marrow disorder, which can be congenital, familial, idiopathic or acquired (Nitsche 2004). Although polycythaemia vera (PV) and primary erythrocytosis (PE) are typically used interchangeably in veterinary medicine, in human medicine PV specifically refers to an acquired myeloproliferative disease secondary to a mutation on the *JAK2* gene (Noumani et al. 2024; Gordeuk et al. 2019). Clinical signs associated with erythrocytosis vary widely depending on the underlying cause and typically arise due to two key pathophysiological features; hypervolemia and hyperviscosity. Common clinical signs include injected mucous membranes, polyuria/polydipsia (PU/PD), ocular changes (e.g., tortuous retinal vessels) and central nervous system signs such as weakness, depression or collapse (Ettinger and Feldman 2016).

A significant sequela of this condition is blood flow stasis caused by increased viscosity, which can lead to hypoxia, vessel rupture and thrombosis (Nitsche 2004; Peterson 1982; Bush and Fankhauser 1972). Both arterial and venous thrombosis are well‐documented in human medicine, with thrombotic or thromboembolic events reported in 39%–41% of cases of PV but are less frequent with other causes of PE (familial and congenital) (Gordeuk et al. 2019; Griesshammer et al. 2019). Although thrombosis is a recognised feature of PV in dogs, it is infrequently reported in the veterinary literature. The first report described a dog euthanised due to a suspected thromboembolic event 18 months after a diagnosis of PE, with post‐mortem findings of a thrombus in the femoral artery (Diogo et al. 2015). The second report described a cerebrovascular thrombotic event in a dog diagnosed with PV (Kay et al. 2018). The only additional canine PV cases identified experienced sudden death with unknown aetiology that could represent thromboembolic disease (Bush and Fankhauser 1972; Meyer et al. 1993). The present case describes an unusual presentation of PE with marked lameness as the chief complaint, discovery of a thrombus on antemortem evaluation and outcome following treatment.

## 2 Case Presentation

A 2‐year‐old male entire Cocker Spaniel with no prior medical history presented to the referring veterinary surgeon (RVS) for progressive right‐hind lameness, which had progressed from a reluctance to jump to intermittent non‐weight bearing of the right pelvic limb, alongside concurrent PD. There was minimal improvement with conservative management (rest) and analgesia (paracetamol 25 mg/kg and codeine 0.5 mg/kg, per‐os, twice daily, Pardale V, Dechra, Northwich, UK). Quantification of the water intake at home was higher than 100 mL per kilogram per day. On follow‐up examination at the RVS 5 days later, haematology revealed a markedly elevated haematocrit of 78% (reference interval: 37%–55%), a normal white blood cell and platelet count, with concurrent appropriate hydration status based on physical examination. Urinalysis identified a urine specific gravity (1.014) consistent with PU and PD, with dipstick results positive for bilirubin, blood and traces of protein. Urine sediment examination and the protein‐to‐creatinine ratio were not performed. Serum biochemistry revealed only a mild elevation in serum bilirubin (19 µmol/L, reference interval: 0–15). Specifically, glucose (5.15 mmol/L, reference interval: 4–8), creatinine (96 µmol/L, reference <125 (International Renal Interest Society 2024)), symmetrical dimethylarginine (SDMA, 10 µg/L, reference <14) and albumin (36 g/L, reference interval: 23–40) were within normal limits, making several metabolic causes of PU/PD unlikely (e.g., diabetes mellitus, chronic kidney disease and liver failure). The dog was then referred out of hours to the Emergency Department of a Teaching Veterinary Hospital for further evaluation.

At the time of referral, the owners reported no history of trauma and, apart from the lameness and PD, had no other clinical complaint. In particular, there was no exercise intolerance or respiratory signs. The dog was up to date with routine vaccines, treated regularly with ecto‐ and endo‐parasiticides, fed a complete commercial diet and had never travelled outside of the United Kingdom. The dog was bright but anxious. Physical examination revealed normothermia, a heart rate of 150 beats per minute, with regular rhythm and absence of murmur on auscultation, moist, dark red mucous membranes and normal capillary refill time (less than 2 s). Peripheral pulses (femoral and dorsal pedal) were fair and synchronous with the heart rate, systolic blood pressure was normal (140 mmHg, oscillometric method), and oxygen saturation was appropriate (98% on pulse oximetry). Abdominal palpation was soft and comfortable, with no findings raising suspicion for masses and no organomegaly. The dog exhibited marked lameness on the right hindlimb, characterised by intermittent off‐loading of this limb, with a reluctance to bear weight during both standing and walking (Video S1, gait upon initial assessment). No wounds or other skin lesions were present on closer examination of the limb. A neurological examination performed by a board‐certified neurologist showed a mild short‐strided hindlimb gait with preferential loading to the left hindlimb (Video S2, initial neurological assessment). The rest of the neurological examination was unremarkable, including normal patellar reflexes bilaterally and intact cutaneous trunci reflexes and withdrawal reflexes in all limbs. No paresis, ataxia or proprioceptive deficits were observed. Orthopaedic examination resulted in similar gait analysis with no other significant finding. Specifically, range of motion of the hip, stifle and tarsal joints was within normal limits without pain on manipulation. No hyperextension or flexion abnormalities were observed. There was normal musculature in all four limbs, no joint effusions or soft tissue swellings and no evidence of patellar luxation.

On the basis of presentation, the problem list included erythrocytosis, right hindlimb gait abnormality/lameness and PU/PD with moderately concentrated urine.

Differential diagnoses of erythrocytosis include relative causes (dehydration and splenic contraction), primary causes (congenital, familial or acquired such as PV) and secondary causes (cardiac, respiratory and renal disorders, including neoplasia). In the present case, dehydration was excluded on physical examination (moist mucous membranes and no skin tent), and splenic contraction was rendered unlikely by the repeatability of the erythrocytosis on multiple occasions. Cardiac and respiratory causes of erythropoietin release were possible, albeit less likely, in the absence of an abnormal respiratory pattern, cough or collapse. Excessive secretion of erythropoietin from renal hypoxia or neoplasm was also possible, although normal serum creatinine made this hypothesis less likely.

Unilateral hindlimb lameness can arise from pain or mechanical dysfunction originating in various anatomical locations, including the skin, long bones, joints, musculotendinous or tendino‐osseous junctions, ligaments, muscles or nerves. In the present case, the absence of external wounds, joint effusion, pain on manipulation or neurological anomalies excluded most of these sites as likely sources of the lameness. Instead, a vascular or neuromuscular aetiology was considered more probable.

The PU/PD was presumed to be secondary to erythrocytosis, which was largely supported by anamnesis and investigations performed by the referring veterinarian (no history of drug administration, no evidence of metabolic or renal disease on blood panels, no electrolyte disturbances and no glucosuria or significant proteinuria on urine dipstick analysis). Remaining hypotheses included nephrogenic diabetes insipidus secondary to a systemic infection, including urinary tract infection and pyelonephritis, cortisol‐related diseases (hypoadrenocorticism and hyperadrenocorticism) or central diabetes insipidus and behavioural, all of which would typically be associated with hyposthenuria.

Initial diagnostic tests included measurement of the packed cell volume (PCV), which was significantly elevated at 90% (reference interval: 37%–55%), and total solids (TS) at 80 g/L (reference interval: 58–73), with haemolysed serum (Figure 1). Venous blood gas and electrolyte analysis (including ionised calcium) were unremarkable, and point‐of‐care ultrasound of the thorax and abdomen revealed no abnormalities. The dog underwent phlebotomy with removal of 160 mL (10 mL/kg) of blood, followed by administration of an equal volume of crystalloids (Hartmann's Lactated Ringers, B. Braun Vet Care, Sheffield, UK). The dog was then hospitalised on intravenous (IV) fluid therapy (Hartmann's 2 mL/kg/h) and an analgesia trial (paracetamol 15 mg/kg IV every 8 h, B Braun, Sheffield, UK; and methadone 0.2 mg/kg IV every 4 h, Comfortan, Dechra, Northwich, UK) until investigations the next morning. On reassessment the following morning, the PCV had dropped to 85% (TS: 60 g/L), prompting a second phlebotomy (20 mL/kg of blood removed but not replaced with crystalloids), following which the PCV reduced to 75%.

**FIGURE 1:**
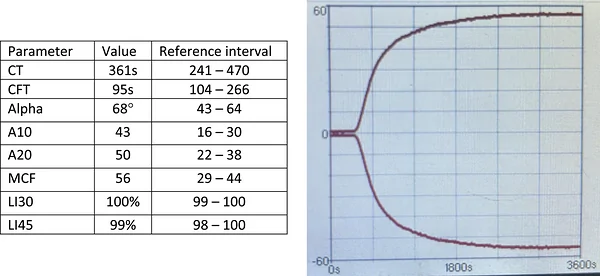
Thromboelastographic trace (viscoelastic coagulation testing) in a dog diagnosed with a right external iliac venous thrombus secondary to primary erythrocytosis after 1 month of treatment with hydroxyurea and rivaroxaban. Parameters outside of the reference interval are in red.

A complete blood count confirmed the elevated haematocrit (70%, reference interval: 39–55), increased haemoglobin (21.7 g/dL, reference interval: 12–18) and revealed moderate hypochromia (mean corpuscular haemoglobin concentration [MCHC] 30.6 g/dL, reference interval: 32–36) along with mild thrombocytopenia (114 × 10^9^/L, reference interval: 200–500) (Table S1). The white blood cell count was within normal limits (7.8 × 10^9^/L, reference interval: 6–15). Blood smear assessment by a board‐certified clinical pathologist showed mild anisocytosis and absence of regenerative features (rare polychromatophils) and confirmed the thrombocytopenia (8.6 platelets per high power field on average, reference interval: 10–15). Further diagnostic tests were undertaken specifically to investigate the underlying cause of erythrocytosis. Arterial blood gas analysis was within normal limits (pO_2_ = 80.6 mmHg, reference interval: 80–100), making cardiorespiratory causes of erythrocytosis less likely. Serum erythropoietin was low (<1.0 mIU/mL, reference interval: 6.6–18.2) making secondary erythrocytosis less likely. Abdominal ultrasound was unremarkable, in particular, with normal kidneys and no evidence of neoplasia or thromboembolic disease. Focal ultrasound of the hindlimb vasculature revealed a thrombus in the right external iliac vein. This thrombus was characterised by focal enlargement of the vein (9 mm wide, normal is approximately 5 mm) with a signal void on colour Doppler extending for approximately one centimetre. B‐mode ultrasound of the vessel lumen appeared normoechoic compared to the surrounding vasculature, consistent with a recent thrombotic event (Figure 2). No adjacent soft tissue changes were apparent. Urinalysis showed moderately concentrated urine (specific gravity 1.018) in keeping with the PD, with traces of blood and protein on dipstick analysis but an inactive sediment. On the basis of these findings, the dog was diagnosed with PE and presumed secondary right external iliac vein thrombus.

**FIGURE 2:**
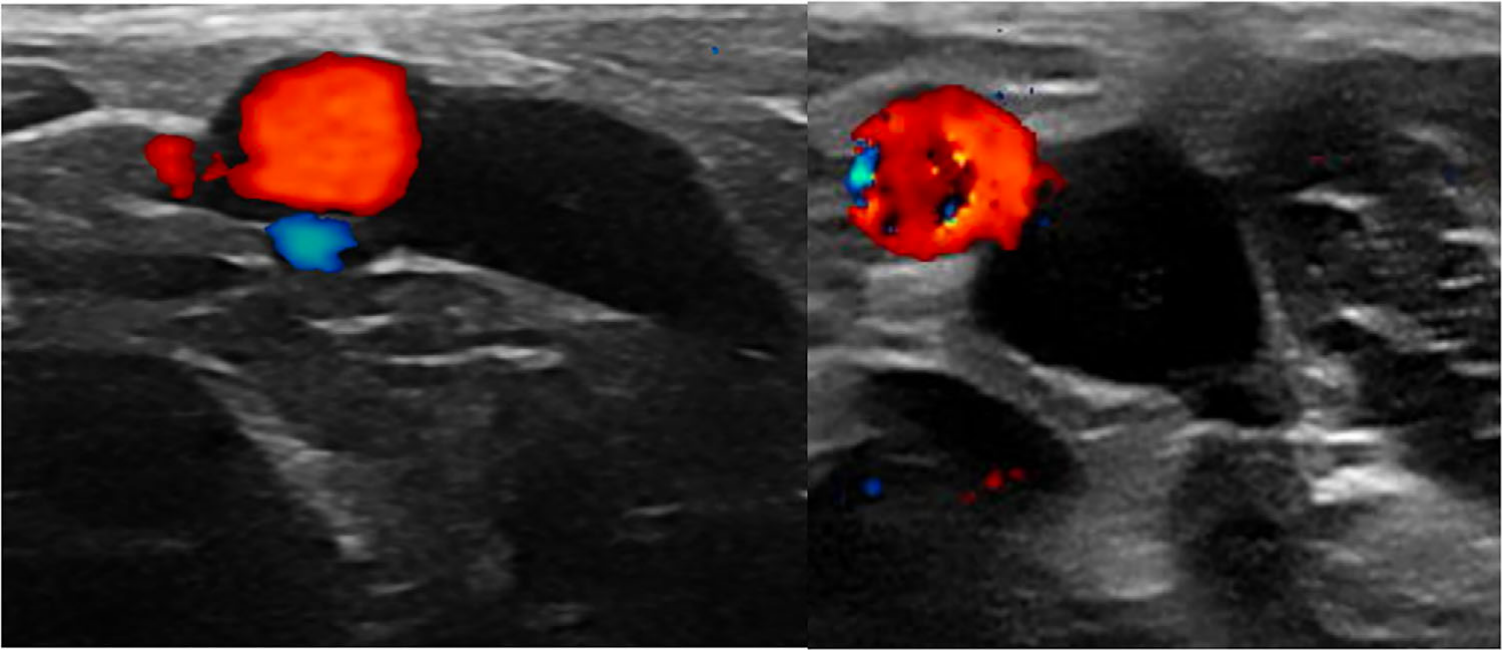
Sagittal (left) and transverse (right) colour Doppler ultrasound short‐axis images showing the common iliac vein bifurcating into the internal iliac vein (near‐field—normal Doppler colour flow) and the external iliac vein (far‐field–absence of Doppler signal). Note the focal luminal enlargement of the external iliac vein on the transverse image and the absence of Doppler signal. Images were acquired with a microconvex array transducer (6–10 MHz) and primary erythrocytosis.

The dog was hospitalised for 3 days on IV fluid therapy and underwent a third phlebotomy (14 mL/kg), with a subsequent reduction in PCV to 68%. Serial blood pressures were moderately to markedly elevated (172–191 mmHg average systolic, oscillometric method), which, in the absence of target organ damage, was suspected secondary to white coat effect. The presumed situational hypertension was managed with anxiolytic medications (trazodone, 100 mg per‐os, three times daily, Flamingo Pharma Ltd, Harrow, UK) and had resolved upon discharge (143 mmHg). Given the presence of a thrombus and the associated risk of further clot formation, antithrombotic therapy was initiated (rivaroxaban 2 mg/kg, per‐os, once daily, Xarelto, Bayer plc, Reading, UK). Cytoreductive therapy for PE was introduced on Day 3 with hydroxyurea (62 mg/kg, per‐os, once daily, Siklos, Addmedica, Paris, France). IV analgesia was gradually tapered, and the dog was discharged with a course of paracetamol (15 mg/kg, per‐os, every 8 h, Crescent Pharma Ltd, Basingstoke, UK), and gabapentin (10 mg/kg, per‐os, every 8 h) for presumed occult pain associated with the lameness. Antithrombotic therapy was continued alongside hydroxyurea for which the dose was decreased to 31 mg/kg after 1 week of treatment.

Two days after discharge, the dog returned to the RVS for a scheduled recheck. The owner reported that the PD had already reduced, and the dog appeared to be improving clinically. The PCV at this visit was 61%. Three weeks later, the PCV had decreased further to 50%, although lameness persisted.

On reassessment at the Teaching Veterinary Hospital after 1 month of treatment, the owner reported continued improvement of both PD and lameness, only describing the right hindlimb as being occasionally held up while standing or eating and mild over grooming of the associated extremity. Physical examination was normal. The PCV was slightly low at 35% (TS: 80 g/L). A complete blood count confirmed the mild anaemia, which was mildly regenerative (reticulocyte count 72.8 × 10^9^/L, reference interval: 20–60; additional haematology parameters are detailed in Table S1). Blood smear assessment by a board‐certified clinical pathologist was suggestive of potential iron‐restricted erythropoiesis (microcytic and hypochromic erythrocytes), with dysplastic changes, and oxidative damage, likely secondary to hydroxyurea administration. Serum biochemistry showed a mildly elevated urea (8.4 mmol/L, reference interval: 1.7–7.4), attributed to hydroxyurea metabolism. Repeat ultrasound of the hindlimb vasculature documented resolution of the previously identified thrombus. Urinalysis showed well‐concentrated urine (specific gravity of 1.050) in keeping with the reported improvement in PD. Dipstick analysis was positive for protein (3+), sediment was inactive, and urine protein‐creatinine ratio was borderline normal (0.2, reference <0.2). Viscoelastic coagulation monitoring (VCM Vet, Entegrion, Durham, USA) suggested ongoing hypercoagulability despite resolution of erythrocytosis and ongoing antithrombotic agent (Figure 1). The mild anaemia with signs of regeneration prompted reduction of hydroxyurea to 31 mg/kg every other day, whereas rivaroxaban was continued unchanged. Two weeks later (7 weeks after initial presentation), the dog was reported to be clinically normal with almost no lameness episodes and a normal thirst. The PCV was still marginally low at 38% (TS: 62 g/L), and haematology revealed marked reticulocytosis (579 × 10^9^/L, reference interval: 10–110), raising concern for gastrointestinal (GI) blood loss as a side effect of hydroxyurea, despite the absence of reported melaena. Consequently, the hydroxyurea dose was reduced to 31 mg/kg every third day and the haemogram normalised. Subsequently, regular revisits with the primary care veterinarian confirmed stable disease; details of serial PCV monitoring and treatment adjustments are summarised in Table 1 and Figure 3. The lameness completely resolved after 3 months of treatment. At the time of writing, 11 months after initial presentation, the dog remains asymptomatic and is maintained on a reduced dose of hydroxyurea (31 mg/kg twice a week) and ongoing daily rivaroxaban.

**TABLE 1:** Packed cell volume (PCV) and medical management over time in a dog diagnosed with a right external iliac venous thrombus secondary to primary erythrocytosis.

Day since presentation	PCV (%)	Hydroxyurea dose	Rivaroxaban dose
0	**90**	None given	None given
1	**85**	None given	2 mg/kg q24 h
2	**75**	None given	2 mg/kg q24 h
3	**68**	62 mg/kg q24 h	2 mg/kg q24 h
6	**61**	62 mg/kg q24 h	2 mg/kg q24 h
21	50	31 mg/kg q24 h	2 mg/kg q24 h
35	**35**	31 mg/kg q24 h	2 mg/kg q24 h
49	**38**	31 mg/kg q48 h	2 mg/kg q24 h
60	47	31 mg/kg Q72 h	2 mg/kg q24 h
90	44	31 mg/kg Q72 h	2 mg/kg q24 h
120	45	31 mg/kg Q72 h	2 mg/kg q24 h
150	44	31 mg/kg 2 times a week	2 mg/kg q24 h

*Note*: Parameters outside the reference interval are shown in bold.

**FIGURE 3:**
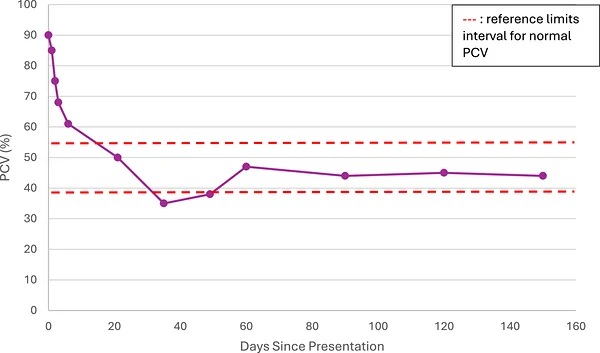
Graphic of the packed cell volume (PCV) trend over time in a dog diagnosed with a right external iliac venous thrombus secondary to primary erythrocytosis and treated with hydroxyurea. The two dotted lines represent the normal PCV reference limits interval.

## 3 Discussion

This report describes venous thrombosis as a suspected cause of non‐orthopaedic lameness in a dog diagnosed with PE.

Thromboembolic diseases are increasingly recognised in small animals and are encountered with a variety of diseases resulting in pro‐coagulable states (Jacinto et al. 2017; Ruehl et al. 2020; Fortuna and Syme 2024; deLaforcade et al. 2022). Although the mechanisms underlying thromboembolism in dogs are not well understood, it is generally accepted that arterial thrombi are platelet‐rich, whereas venous thrombi are predominantly composed of cross‐linked fibrin (Konečný 2010). Lameness secondary to thrombi has previously been described, more commonly in association with arterial thrombosis linked to underlying conditions such as hyperadrenocorticism, neoplasia, trauma, heartworm, protein‐losing nephropathy, and cardiac disease (Boswood et al. 2000; Andruzzi et al. 2021; Ahn et al. 2019). Lameness secondary to venous thrombi is reported rarely in the veterinary literature and is limited to four cases (Jaffe et al. 1999; Calvo‐Sánchez et al. 2024; Teshima et al. 2008; Grösslinger et al. 2004). The first described a dog with extensive thrombosis of the caudal vena cava, right external iliac vein, right femoral vein and right common iliac vein secondary to adrenocortical carcinoma invading the vena cava and presenting with bilateral hindlimb weakness and oedema. The second described a dog with leishmaniasis that developed bilateral hindlimb lameness after the implementation of meglumine antimoniate and allopurinol treatment. The patient was diagnosed with a large venous thrombus of the caudal vena cava and external iliac veins presumably secondary to the high levels of circulating immunoglobulins. The third case presented with pyrexia and unilateral non‐weight‐bearing lameness of the right hindlimb with associated swelling, secondary to an iliopsoas abscess that predisposed to ipsilateral iliac and femoral vein thrombosis. The last case described a dog with pituitary‐dependent hyperadrenocorticism presenting with chronic bilateral hindlimb lameness and pain and diagnosed with thrombosis of the caudal vena cava and abdominal aorta. To the authors' knowledge, this is the first reported case of lameness associated with venous thrombus in a dog diagnosed with PE.

Orthopaedic causes were not definitively ruled out as neither skeletal radiography, computed tomography or arthrocentesis was performed. Immune‐mediated polyarthritis and other joint and long bone pathologies remain possible differentials. However, the lack of joint pain on manipulation or palpable joint effusion and the absence of systemic signs of inflammation (e.g., fever and leukocytosis) made these less likely. Similarly, computed tomography would have been a valuable tool to further assess the extent of thrombotic disease but was not pursued due to costs. Muscular disease was also considered less plausible due to the absence of muscular atrophy or pain on manipulation. Ultimately, the identification of an iliac venous thrombus and the resolution of the lameness (and thrombus) after anticoagulant therapy support a vascular cause. Even so, the inability to completely exclude muscular or orthopaedic disease remains a limitation of this case. We acknowledge additional limitations in the diagnostic work‐up. A urine protein to creatinine ratio was not performed at initial presentation, which would have provided a more complete assessment of renal health, but significant proteinuria was deemed unlikely in a dog with trace protein on dipstick and a urinary specific gravity of 1.018 (Zatelli et al. 2010). Electromyography was not conducted, limiting the ability to rule out neuromuscular disease as a cause of lameness. Thoracic radiographs and echocardiography were not performed as normal aortic blood gas and point‐of‐care ultrasound made significant cardiorespiratory disease unlikely, but this precluded the assessment of potential secondary cardiac complications such as myocardial hypertrophy or other structural heart changes.

Unlike acute thrombotic events, which are more often arterial in origin and typically cause severe pain and marked limb dysfunction (Andruzzi et al. 2021; Calvo‐Sánchez et al. 2024), this case presented with more chronic, progressive clinical signs. Chronic thromboemboli, both arterial and venous, are known to cause waxing and waning lameness, exercise intolerance and intermittent pain (Jaffe et al. 1999; Gonçalves et al. 2008), and this presentation underscores the importance of including vascular causes in the differential diagnoses for dogs presenting with chronic lameness. Notably, pain was not a prominent feature on repeated clinical examinations despite marked lameness. In human medicine, post‐thrombotic syndrome is variably associated with pain, which is not reported to be elicited on palpation, and management does not primarily rely on analgesia but instead focuses on exercise, limb elevation and compression therapy (Giannoukas et al. 2008; Baldwin et al. 2013). Importantly, the absence of pain on palpation in this case does not preclude the presence of neuropathic or discomfort, which may manifest differently on clinical examination.

Neurologic complications following venous thromboembolism (VTE) are thought to arise through various mechanisms, including compression of adjacent nerves caused by vascular distension at the site of the thrombus (Salgado‐Parente et al. 2023). This mechanical effect, along with local increases in pressure, can lead to nerve entrapment or injury (Zhang et al. 2024; Bayramoglu et al. 2014). Additionally, inflammation associated with thrombophlebitis may extend to perineural tissues, contributing to pain and neurologic dysfunction. In contrast, arterial thromboembolism can result in ischaemia of peripheral nerves, potentially causing axonal degeneration and demyelination—manifesting clinically as paresis, reduced reflexes and sensory deficits (Andruzzi et al. 2021; Giannoukas et al. 2008; Baldwin et al. 2013; Schneck 2021). In the present case, the absence of features such as cold extremities or weakened peripheral pulses suggested a venous rather than arterial thrombotic event, as confirmed on ultrasound. However, the marked unilateral lameness could have reflected peripheral nerve involvement and pain. In ‘post‐thrombotic syndrome’ in humans, a recognised sequelae of VTE, often characterised by ongoing or intermittent pain as well as altered sensations such as tingling or numbness in the affected limb. Although such subjective sensations cannot be directly assessed in dogs, in the present case, over grooming of the right hindlimb extremity was described despite confirmed resolution of the thrombus on ultrasound, and these signs may be consistent with neuropathic discomfort (Baldwin et al. 2013).

The mechanisms underlying thrombosis in erythrocytosis have been primarily explored in human medicine. One key contributor is the elevated red blood cell mass, which increases viscosity and promotes blood stasis, contributing to thrombus formation. Additionally, the increased cellular mass can displace the platelets towards the vessel wall, increasing their contact with endothelial surfaces and subendothelial components like collagen and von Willebrand factor, thereby promoting platelet adhesion and thrombus formation (Kroll and Michaelis 2015). The clinical significance of blood viscosity as a thrombotic driver is supported by evidence from clinical trials in human PV patients, which demonstrated that maintaining a normal haematocrit significantly reduces the risk of major thrombotic events and cardiovascular mortality (Marchioli et al. 2013). However, individuals with secondary erythrocytosis (e.g., chronic pulmonary disease) do not always display these prothrombotic tendencies (Hasselbalch et al. 2021), suggesting that increased viscosity alone does not fully account for increased thrombogenesis in PV. In addition to haematological changes, a common genetic mutation, *JAK2* V617F, is known to play a central role in PV‐associated thrombosis in humans. Patients with this mutation exhibit changes that promote a hypercoagulable state, including, though not limited to, increased platelet activation markers, elevated inflammatory cytokine production and altered endothelial cell adhesion (Marchioli et al. 2013; Hasselbalch et al. 2021). Although less extensively studied in veterinary medicine, the *JAK2* V617F mutation has been identified in at least one dog with suspected primary polycythaemia, suggesting that similar pathogenic mechanisms may be shared between canine and human PV (Beurlet et al. 2011). Moreover, thromboembolic events have also been reported in other forms of PE, such as congenital erythrocytosis, indicating that the prothrombotic risk in PE is not exclusive to PV (Mallik et al. 2021). Further research is needed to clarify the genetic and molecular drivers of thrombosis in dogs with these conditions.

The updated CURATIVE (Consensus on the Rational Use of Antithrombotics in Veterinary Critical Care) guidelines for antithrombotic and thrombolytic therapy in veterinary medicine do not list polycythaemia as a condition warranting routine antithrombotic therapy (deLaforcade et al. 2022). This contrasts with human guidelines, which recommend prophylactic low‐dose aspirin for patients with PV who are at low risk of bleeding (Squizzato et al. 2013; Bachleitner‐Hofmann et al. 2003; McMullin et al. 2019). Patients considered at high risk of bleeding are those with extreme thrombocytosis (risk of developing acquired von Willebrand syndrome), a history of major haemorrhage, known GI pathology such as ulcers, advanced liver disease and those taking concurrent antiplatelet/coagulant therapy. In these high‐risk patients antiplatelet and anticoagulant therapy is used with caution, often weighing up benefit‐risk and using the lowest possible doses. The use of prophylactic antithrombotic therapy in veterinary patients with PV is limited to a case report in which heparin was unsuccessfully used to treat a suspected thromboembolic event in a dog (Diogo et al. 2015) and hypothesised use of aspirin in scientific reviews describing potential treatments of the disease (Nitsche 2004).

In the present case, identification of a thrombus in the iliac vein prompted the initiation of rivaroxaban. Rivaroxaban is a direct oral anticoagulant (DOAC) and is specifically a direct factor Xa inhibitor. It works by disrupting the coagulation cascade, halting the conversion of prothrombin to thrombin and therefore blocking fibrin clot formation (Uchida et al. 2020). Rivaroxaban was used over antiplatelet therapy like aspirin or clopidogrel due to the presence of a venous thrombus rather than an arterial. This is due to venous clots being predominantly made up of red blood cells and fibrin, whereas arterial clots are platelet‐dense (deLaforcade et al. 2022). Additionally, multiple recent studies and case reports demonstrate that rivaroxaban use in dogs with suspected or confirmed thromboembolism leads to partial or complete clot resolution in a substantial number of cases, whereas such clinical outcomes are absent in comparable veterinary scenarios when aspirin is used (Uchida et al. 2020; Griebsch et al. 2022; Conversy et al. 2017; Morassi et al. 2016; Tracy et al. 2022).

At the 1‐month recheck, VCM revealed persistent hypercoagulability. Although the iliac thrombus had resolved, this finding supported the continued administration of rivaroxaban. Unfortunately, no further follow‐up visits were conducted at the Teaching Veterinary Hospital, and additional thromboelastography or coagulation monitoring was not performed. This raises important questions regarding the appropriate duration of antithrombotic therapy and the optimal methods for monitoring coagulation status. In human medicine, VTE is typically treated with anticoagulation for a minimum of 3 months, with extended or indefinite therapy recommended in the presence of persistent pro‐thrombotic risk factors, such as myeloproliferative disorders (Weitz et al. 2020). There are studies looking at therapeutic monitoring of rivaroxaban use which conclude that gold standard monitoring is anti‐Xa assay when available, but prothrombin time (PT) and viscoelastic testing are practical and valid alternatives (Phillips et al. 2025). A recent study demonstrated that an increase of 1.5–1.9 times from baseline PT value (measured about 3 h after dosing) generally corresponds to a therapeutic anticoagulant effect, with PT measurement being a more cost‐effective and accessible form of monitoring (Phillips et al. 2025).

Therapy should be discontinued when the risk of thrombosis resolves, adverse effects emerge or ongoing risk no longer justifies treatment (e.g., using both laboratory and clinical data to guide decision‐making). Given the documented risk of thrombosis and thromboembolic events, which can lead to death in both canine patients and humans with PV, the potential for prophylactic use of antithrombotics long‐term in these cases warrants further investigation. Reducing haematocrit alone to improve blood stasis and mitigate its consequences may not be sufficient. This case demonstrated that the dog remained in a presumptive hypercoagulable state (based on VCM, with no additional tests for hypercoagulability performed) even after several weeks of achieving a normal haematocrit alongside rivaroxaban administration, highlighting the need for continued evaluation and monitoring in similar cases.

Hydroxyurea is a critical drug for the management of PV. It is a chemotherapeutic agent and a member of the alkylating group of drugs, primarily functioning as an inhibitor of DNA synthesis, resulting in bone marrow suppression. It stops the production of erythroid precursors and, in turn, lowers the circulating red cell volume. The use and dosing of hydroxyurea in PV cases have been variable in previous reports, ranging from 15 to 60 mg/kg every 24–72 h (Nitsche 2004; Peterson 1982; Bush and Fankhauser 1972; Diogo et al. 2015; Kay et al. 2018; Phillips et al. 2025; Gray et al. 2003; Holopainen et al. 2022; Watson and Yeats 1984; Billström‐Saxon and Giger 2024; Quesnel and Kruth 1992). When appropriately dosed and monitored, the drug is generally well tolerated in dogs; however, side effects can occur, and dose adjustments are often necessary. The most common adverse effects include myelosuppression (anaemia, leukopenia and thrombocytopenia), GI disturbances and dermatological changes such as onychomadesis and ulceration—though the latter are reported mostly in humans. Most side effects are mild and reversible with dose reduction. In the present case, the dog was initially started at the higher end of the dose range, and this quickly resulted in mild anaemia. Despite dose reduction, the anaemia persisted and temporarily worsened. At this point the haematocrit had dropped and there was a significant increase in reticulocytes, which raised concerns for concurrent GI bleed. In humans, there are rare reports of hydroxyurea causing GI bleeding as a result of myelosuppression and thrombocytopenia. A further dose reduction resolved this anaemia. GI bleeding was never confirmed, and the platelet count remained normal; however, a faecal occult blood test could have been performed if the suspicion persisted. The final hydroxyurea regimen was 31 mg/kg twice weekly, which has never been reported in the literature with most cases requiring 15 mg/kg every 1–2 days to prevent relapse (Nitsche 2004; Peterson 1982; Bush and Fankhauser 1972; Diogo et al. 2015; Kay et al. 2018; Giannoukas et al. 2008; Baldwin et al. 2013; Schneck 2021; Squizzato et al. 2013; Bachleitner‐Hofmann et al. 2003).

Although not typically reported in humans, PU and PD are well‐recognised and prominent clinical signs in dogs with PV (1). The mechanism is multifactorial and not fully understood but is most commonly attributed to blood hyperviscosity leading to impaired renal perfusion, disruption of urine‐concentrating mechanisms and altered vasopressin (antidiuretic hormone) release (2). These abnormalities often resolve with normalisation of the haematocrit. In the present case, alternative causes of PU/PD such as hypoadrenocorticism, hyperadrenocorticism, central diabetes insipidus and behavioural PD were not fully excluded. However, further investigation was deemed unnecessary given the high likelihood that erythrocytosis was the underlying cause, a conclusion further supported by the resolution of PU/PD following treatment.

## 4 Conclusion

This case describes an unusual presentation of PE in a dog, manifesting as unilateral lameness subsequent to VTE. This is not previously described in the veterinary literature and highlights the importance of considering vascular disorders as a differential diagnosis for lameness. It also raises questions regarding the necessity of prophylactic antithrombotic therapy in dogs diagnosed with PE and highlights the limited data available regarding the monitoring of long‐term antithrombotic treatment.

## 5 Clinical Relevance

In dogs presenting with unexplained non‐orthopaedic and non‐neurological lameness, particularly when accompanied by marked erythrocytosis, clinicians should consider vascular thrombosis as a potential underlying cause. Even in the absence of classical signs of arterial thromboembolism, targeted vascular imaging and coagulation assessment may be warranted. Management should focus not only on reduction of haematocrit to alleviate hyperviscosity but also on appropriate antithrombotic therapy when thrombosis is identified or strongly suspected, as normalisation of haematocrit alone may not fully mitigate thrombotic risk.

## Author Contributions

**Maria Lazaro Munoz**: investigation. **Robyn Belch**: conceptualisation, investigation, writing – original draft. **Aran Nagendran**: conceptualisation, investigation, writing – review and editing, data curation, supervision. **Efa Llewellyn**: conceptualisation, investigation. **Ingrid Isaac**: investigation, writing – review and editing, data curation. **Albert Aguilera Padros**: investigation, writing – review and editing. **Perrine Henry**: writing – original draft, writing – review and editing, data curation, supervision, conceptualisation, investigation. **Ben Lloyd‐Bradley**: investigation. **Marisa Ferreira**: writing – review and editing, data curation, supervision, investigation.

## Funding

The authors have nothing to report.

## Ethics Statement

The authors confirm that the ethical policies of the journal, as noted on the journal's author guidelines page, have been adhered to. No ethical approval was required as this is a review article with no original research data.

## Conflicts of Interest

The authors declare no conflicts of interest.

## Supporting information




**Table S1**: Detailed haematology parameters in a neutered male dog diagnosed with primary erythrocytosis and treated with hydroxyurea.

**Videos 1**: Gait/neurological examination on initial presentation.

**Videos 2**: Gait/neurological examination on initial presentation.

## Data Availability

The data that support the findings of this study are available on request from the corresponding author. The data are not publicly available due to privacy or ethical restrictions.
