# Supplementary material for: Primary Erythrocytosis (Polycythaemia) in a Neutered Male Dog Presenting With Right Hindlimb Lameness
Source: Vet Med Sci. 2026 Nov 3;12(6):e71320. doi: 10.1002/vms3.71320 (PMC13731734; doi:10.1002/vms3.71320)
Supplement: Supplementary file 1 [file VMS3-12-e71320-s003.docx]

Supplementary Table 1 : Detailed haematology parameters in a neutered male dog diagnosed with primary erythrocytosis and treated with hydroyrurea.

| **Timeline** | **HCT**  **(%)** | **HGB**  **(g/dL)** | **RBC**  **(x10^12^/L)** | **MCV**  **(fL)** | **MCH**  **(pg)** | **MCHC**  **(g/dL)** | **RDW**  **(%)** | **PLT**  **(K/µL)** | **Hydroxyurea dose** |
| --- | --- | --- | --- | --- | --- | --- | --- | --- | --- |
| Before referral | **78.5** | **23** | **14.7** | **53.4** | **15.7** | **29.3** | **21.8** | **121** | None given |
| Initial presentation* | **70** | **21.7** | **11.19** | 63.3 | N/A | **30.5** | 30.1 | **114** | None given |
| + 3 days | **59** | 18.3 | **10.98** | **53.5** | **16.7** | 31.2 | **21.9** | 225 | 62 mg/kg q24hr |
| + 45 days | **32.5** | **11.4** | **4.63** | 70.1 | 24.7 | 35.2 | **19.5** | 343 | 31 mg/kg q48hr |
| + 60 days* | **57** | 15.4 | 5.78 | **98.4** | N/A | **27.1** | N/A | 356 | 31 mg/kg q72 hr |
| + 90 days | 45 | 16.2 | 6.5 | 69 | 25 | 36.2 | 16.4 | 218 | 31 mg/kg q72 hr |
| + 120 days | 45 | 16 | 6.91 | 65.5 | 23.1 | 35.3 | 16.5 | 229 | 31 mg/kg q72 hr |
| + 150 days | 44 | 15.3 | 6.09 | **72.2** | 25.1 | 34.8 | 16.2 | 288 | 31 mg/kg 2 times a week |

Abnormal results are in bold. HCT values are those reported by the automated haematology analyser and therefore may differ from the PCV values presented in Table 1.

Abbreviations and reference intervals in bracket:

**RVS:** referring veterinary surgeon; **HCT:** haematocrit [36.6 – 54.5]; **HGB:** haemoglobin [12.2 – 18.4]; **RBC:** red blood cell count [5.83 – 9.01]; **MCV:** mean corpuscular volume [55.8 – 71.6]; **MCH:** mean corpuscular haemoglobin [17.8 – 28.8]; **MCHC:** mean corpuscular haemoglobin concentration [30.9 – 38.6]; **RDW:** red cell distribution width [14.7 – 17.9]; **PLT:** platelet count [175 – 500]; **N/A:** not available.

All complete blood counts were performed using a ProCyte Dx Haematology Analyzer (IDEXX Laboratories, Westbrook, ME, USA), except for the two samples marked with an asterisk (*), which were analysed at a referral laboratory.
